# 573 Interfacility Transfers for Burn Patients with Concomitant Traumatic Injuries

**DOI:** 10.1093/jbcr/irac012.201

**Published:** 2022-03-23

**Authors:** Kevin D Kirkland, Austin Gratton, Caleb Mentzer, John Kubasiak, James R Yon

**Affiliations:** Novant Health/New Hanover Regional Medical Center, Wilmington, North Carolina; Novant Health/New Hanover Regional Medical Center, Wilmington, North Carolina; Spartanburg Regional Healthcare System, Spartanburg, South Carolina; Loyola University Medical Center, Maywood, Illinois; Novant Health/New Hanover Regional Medical Center, Wilmington, North Carolina

## Abstract

**Introduction:**

Burn patients with concomitant traumatic injuries suffer increased morbidity and mortality. Complex care coordination for these patients often requires interfacility transfers. We sought to examine the outcomes for traumatically injured burn patients to identify the occurrence and associated reasons for trauma system transfers in this group.

**Methods:**

The National Trauma Data Bank was examined from years 2007 to 2016 for 6,565,577 patients with traumatic, burn, and concomitant burn & traumatic injuries to evaluate demographic data, ED and hospital dispositions, length of ICU stay, ventilator days, mortality, and interfacility transfers. There were 5,068 patients with both traumatic and burn injuries, 145,890 patients with burn injuries, and 6,414,619 patients with traumatic injuries. Non-parametric Kruskal Wallis, analysis of variance, chi-square, Mann Whitney, and T-tests were utilized for comparisons.

**Results:**

Patients were 69.1% male in the trauma/burn, 67.1% male in the burn, and 62.2% male in the trauma groups (P< 0.001). Trauma/burn patients were more often admitted to the ICU from the ED at 35.5% compared to 27.1% for burn and 19.4% for trauma (P< 0.001). ICU stay was longer for trauma/burn patients at 5.00 median days (IQR 2.00, 15.00) versus 3.00 (IQR 1.00, 10.00) for burn and 3.00 (IQR 1.00, 5.00) for trauma (P< 0.001). Trauma/burn patients had more ventilator days at 3.00 median days (IQR 1.00, 11.75) compared to 2.00 (IQR 1.00, 9.00) for burn and 2.00 (IQR 1.00, 7.00) for trauma (P< 0.001). Trauma/burn patients had increased mortality at 4.9% versus 2.5% for burn and 3.2% for trauma (P< 0.001). For hospital disposition, trauma/burn patients required more interfacility transfers 2.5% compared to 1.7% for burn and 1.3% for trauma (P< 0.001). For level 1 trauma centers, 5.5% of trauma/burn, 7.1% of burn, and 0.5% of trauma patients required interfacility transfers. For level 2 trauma centers, 29.1% of trauma/burn, 47.0% of burn, and 2.8% of trauma patients required interfacility transfers.

**Conclusions:**

Among level 1 and level 2 trauma centers, patients with only burns and burn patients with concomitant traumatic injuries required more interfacility transfers, and level 2 trauma centers required more interfacility transfers for all patients.

